# Effects of Polyoxymethylene Fiber on Fresh and Hardened Properties of Seawater Sea-Sand Concrete

**DOI:** 10.3390/polym14224969

**Published:** 2022-11-16

**Authors:** Xuanyi Xue, Fei Wang, Jianmin Hua, Neng Wang, Lepeng Huang, Zengshun Chen, Yunhang Yao

**Affiliations:** 1 School of Civil Engineering, Chongqing University, Chongqing 400045, China; 2 Key Laboratory of New Technology for Construction of Cities in Mountain Area, Chongqing University, Ministry of Education, Chongqing 400045, China; 3 School of Management Science and Real Estate, Chongqing University, Chongqing 400045, China

**Keywords:** seawater sea-sand concrete, polyoxymethylene (POM) fiber, early-age cracking performance, workability, mechanical property

## Abstract

Seawater and sea sand are used in concrete to reduce the consumption of freshwater and river sand. To improve the mechanical properties and cracking resistance of concrete, polymer fiber is commonly used. In this study, polyoxymethylene (POM) fiber was innovatively applied to seawater sea-sand concrete (SWSSC), and the workability, early-age cracking behavior, and mechanical properties of SWSSC reinforced with POM fiber were investigated experimentally. A total of 6 kinds of SWSSC mixtures and 72 specimens were included. The test results indicated that with increases in fiber volume fractions (*ρ*), the workability of SWSSC decreased correspondingly. Compared with plain SWSSC, for SWSSC with *ρ* = 1%, the decreases in slump and expansibility were 110.6 and 91.9 mm, respectively. POM fiber had a significant enhancing effect on the early-age cracking resistance of SWSSC. Compared with those of plain SWSSC, the cracking indices *a_c_*, *b_c_*, and *c_c_* of the POM-1 specimen decreased by 77.0%, 89.4%, and 97.6%, respectively. Cube and axial compressive tests, splitting tensile tests, and flexural tests were conducted to clarify the effects of POM fiber on the mechanical properties of SWSSC. Compared with plain SWSSC, SWSSC with POM fiber performed better in terms of mechanical properties. Predictive equations were proposed to quantify the effects of POM fiber on the mechanical properties of SWSSC. The failure performances of the SWSSC specimens were discussed and their complete stress–strain curve was analyzed. A stress–strain model for SWSSC was suggested. According to the model, the complete stress–strain curve of SWSSC with any POM fiber content could be determined.

## 1. Introduction

Steel and concrete exhibit good coordination in their working performance, because the linear thermal expansion coefficient of steel is similar to that of concrete. Considering their outstanding resistance and convenient construction, reinforced concrete (RC) structures are widely used, such as bridges, dams, or industrial and civil structures [1,2]. River sand and freshwater are raw materials used in ordinary concrete. With the acceleration of urbanization, concrete is being consumed in large quantities, resulting in the large-scale exploitation of river sand and freshwater [3]. On the one hand, the massive exploitation of river sand destroys the aquifers under riverbeds and causes large amounts of surface river water to leak into the ground, causing the river water to dry up. On the other hand, the massive exploitation of river sand makes the river channel unable to discharge normally during the flood season. The riverbank cannot protect the coast, which may cause large fields to be flooded and soaked. Furthermore, large amounts of freshwater are consumed, aggravating water shortages. Therefore, the high consumption of concrete seriously affects the natural environment [4]. To balance the development of engineering construction and the protection of the natural environment, it is important to find alternative replacements for river sand and freshwater. Seawater and sea sand are commonly considered as alternative solutions for the sand and water used in concrete [3,5]. The natural resources of seawater and sea sand are much more abundant than those of freshwater and river sand [6,7]. The usage of seawater and sea sand instead of freshwater and river sand in concrete can mitigate the abovementioned environmental problems. The use of local seawater and sea sand in island reef construction can reduce the transportation costs of raw materials and, thus, the engineering costs [5,8]. However, it is worth noting that the use of untreated seawater and sea sand in concrete brings large amounts of chloride. The chloride in untreated seawater and sea sand penetrates the passive film on the reinforcing steel in concrete, resulting in serious corrosion [9,10,11,12,13,14,15]. This, in turn, reduces the resistance of RC structures. It is worth noting that the above corrosion issue could be solved by using non-corrosive reinforcement, such as stainless steel bars [16,17], fiber-reinforced polymer (FRP) bars [18,19], and/or bimetallic steel bars [20,21,22,23,24,25,26,27,28]. Corrosion-resistant steel [29,30,31,32] also makes it possible to use seawater and sea sand in steel–concrete composite structures. Concrete made using untreated seawater and sea sand has gradually become a research hotspot in recent years [33,34,35]. Many researchers have conducted studies on the aforementioned seawater sea-sand concrete (SWSSC) [4,6,36,37]. Experimental results have indicated that the workability and mechanical properties of SWSSC are similar to those of ordinary concrete made using freshwater and river sand. Therefore, it is possible to use SWSSC in RC structures as a replacement for traditional concrete. Based on the current research on SWSSC [3,4,6,34], the noteworthy uncertainty in the chemical composition of seawater and the fineness modulus of sea-sand can be observed, leading to differences in the performance of SWSSC. Related studies are still needed to support the engineering application of SWSSC properly.

Fiber-reinforced composites are advanced materials. The use of fiber can significantly improve the crack resistance of concrete. With the deepening of fiber research and the improvement of fiber production processes, fiber is increasingly used in concrete to improve its mechanical properties [38,39]. Tensile properties are important for concrete, which can be effectively enhanced by polymer fibers [40,41,42]. Saradar et al. [43] studied the crack resistance effects of steel fiber, glass fiber, basalt fiber, polypropylene fiber, and polyolefin fiber on concrete, and found that the addition of fiber increases the compressive strength by 16% and 20% at the age of 3 and 7 days, respectively. Alwesabi et al. [44] found that micro steel fiber and polypropylene fiber improved the mechanical properties of concrete. Furthermore, cracks in the concrete had a significant effect on the durability of RC structures [36]. To improve the crack resistance of concrete, polymer fiber is an alternative solution. Polyoxymethylene (POM) fiber is a new kind of polymer fiber that presents outstanding alkali resistance and tensile properties [45]. POM fiber exhibits different mechanical properties from other fibers [46]. According to previous studies, the tensile strength of POM fiber is higher than that of polypropylene fiber, and the ductility of POM fiber is higher than that of glass fiber [35,47,48]. Although steel fiber has good tensile properties and ductility, the large amounts of chloride ions in seawater sea-sand concrete may cause corrosion of steel fiber. In conclusion, POM fiber is more suitable for seawater sea-sand concrete than other fibers. To the best of the authors’ knowledge, there has been little investigation of SWSSC reinforced with POM fiber. The effects of POM fiber on the early-age cracking performance and mechanical properties of SWSSC are still not clear.

To clarify the aforementioned issue, fundamental studies of SWSSC reinforced with POM fiber were conducted to support its widespread application in actual structures. Experimental investigations of the workability, early-age cracking performance, and mechanical properties of SWSSC reinforced with POM fiber were performed. Details of the materials and test methods used are introduced in Section 2. Based on references in the code, workability tests, early-age cracking tests, cube and axial compressive tests, splitting tests, and flexural tests were conducted in this study. The results of the above tests for different mechanical properties are introduced in Section 3. Based on the test results, predictive equations were proposed to quantify the effects of POM fiber on the mechanical properties of SWSSC, and a stress–strain model for SWSSC was suggested. 

## 2. Experimental Program

### 2.1. Raw Materials

#### 2.1.1. Seawater and Sea Sand

It is worth noting that the seawater and sea sand used in the SWSSC experiment were all untreated. The seawater was taken from Quanzhou (118.36° E, 24.56° N) in Fujian Province, China. The chemical composition of the seawater is shown in Table 1. Compared with those of tap water, the ion contents of the seawater were much higher—particularly the Cl^−^ and Na^+^. The sea sand used in the test was taken from Guangzhou (113.17° E, 23.79° N) in Guangdong Province, China. Before the specimens’ production, the sea sand was dried in the Sun to control its moisture content. Then, a screening test was conducted to determine the fineness modulus (*M_x_*) of the untreated sea sand. The screening test standard of the sand was in accordance with JGJ 52-2006 [49]. The *M_x_* was determined through Equation (1), where *A*_1_, *A*_2_, *A*_3_, *A*_4_, *A*_5_, and *A*_6_ denote the cumulative percentages of sieve residues for sieves with diameters of 4.75, 2.36, 1.18, 0.6, 0.3, and 0.15 mm, respectively. The test results are shown in Table 2. After calculation, it turned out that *M_x_* = 2.41. The sea sand fitted the requirements of the medium sand category, indicating that the sea sand could be used in the production of SWSSC specimens.

(1) $$M_{x}=\frac{\left(A_{2}+A_{3}+A_{4}+A_{5}+A_{6}\right)-5A_{1}}{100-A_{1}}$$

#### 2.1.2. POM Fiber

**Figure 1:**
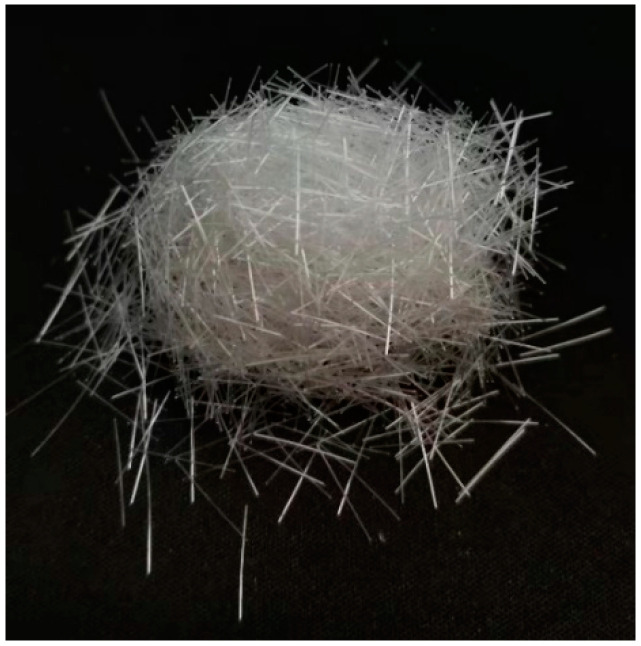
POM fiber used in this study.

The POM fiber was used in this study to improve the early-age cracking resistance and mechanical properties of SWSSC. The physical properties of the POM fiber were tested by Yuntianhua Co., Ltd., Yunnan, China, as shown in Table 3. As shown in Figure 1, the diameter and length of the POM fiber were 0.2 mm and 12 mm, respectively. The POM fiber exhibited outstanding alkali resistance, indicating that it has wide applicability in concrete structures. Furthermore, the POM fiber used in this study did not easily agglomerate, which reduced the difficulty of construction.

### 2.2. Mixture Proportions and Specimen Preparation

For the experimental investigation in this study, a total of six kinds of SWSSC mixtures were included, as shown in Table 4. NF was the control group, which incorporated no fiber in the mixture. POM-0.2 denotes that the POM fiber volume fraction (*ρ*) was 0.2%. Specimens were prepared under the same laboratory conditions. We weighed the sea sand, coarse aggregates, and cement according to the mix proportions, poured them into the mixer, and dry-mixed them for 1 min. After that, we added seawater and admixture into the mixer and mixed for a further 1 min. Finally, POM fiber was evenly sprinkled into the concrete mixture, and mixing continued for another 2 min. This operation ensured that the POM fiber was evenly distributed in the concrete. After mixing, the early-age cracking behavior and workability of the SWSSC were investigated. As for the test specimens used for the mechanical properties tests, a vibration table was used to compact the SWSSC in molds. The specimens were removed from the molds one day after casting. Then, all of the specimens were cured in a standard curing room for 28 d at 20 ± 2 °C and 95% relative humidity. For each kind of mechanical test, 18 SWSSC specimens were prepared, as shown in Table 5. A total of 72 test specimens were included in this mechanical performance investigation.

### 2.3. Test Method

#### 2.3.1. Workability Test

**Figure 2:**
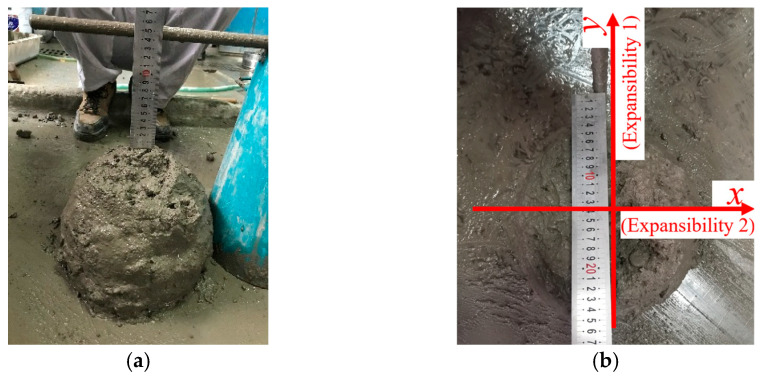
Workability test (*ρ* = 0.6): (**a**) slump test; (**b**) expansibility test.

The workability of concrete reflects the consistency of the mixture, which directly affects the concrete’s quality and construction difficulty. To quantify the workability of different mixtures, slump and expansibility tests were performed, as shown in Figure 2. All W/C ratios of all SWSSC mixtures were controlled to be the same. According to GB/T 50080-2016 [50], a standard slump cone was used to measure the slump and expansibility of concrete. The expansibility was the average of expansibility 1 and expansibility 2.

#### 2.3.2. Early-Age Cracking Test

**Figure 3:**
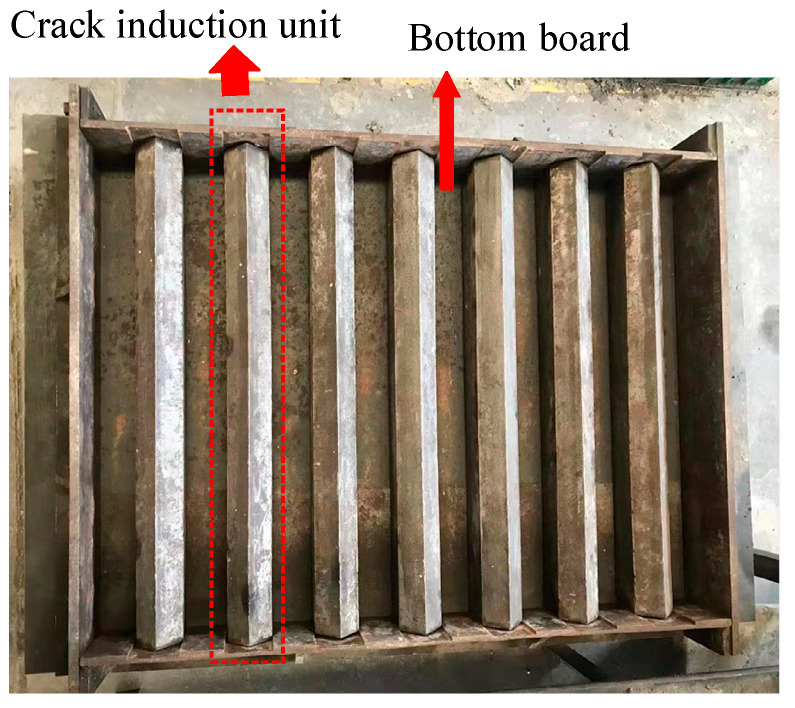
Early-age cracking test instrument.

To investigate the early-age cracking performance of SWSSC with different *ρ*, the knife-edge binding method recommended in GB/T 50082-2009 [51] was selected. The dimension of the test specimen was 800 mm × 600 mm × 100 mm, as shown in Figure 3. Seven crack induction units were distributed on the steel bottom board. Thirty minutes after casting, the wind speed at 100 mm above the center of the test specimen was controlled to be 5 m/s. The direction of the wind was parallel to that of the crack induction units. After 24 h of mixing with seawater, the cracking performance of the test specimen was recorded.

#### 2.3.3. Mechanical Performance Test

**Figure 4:**
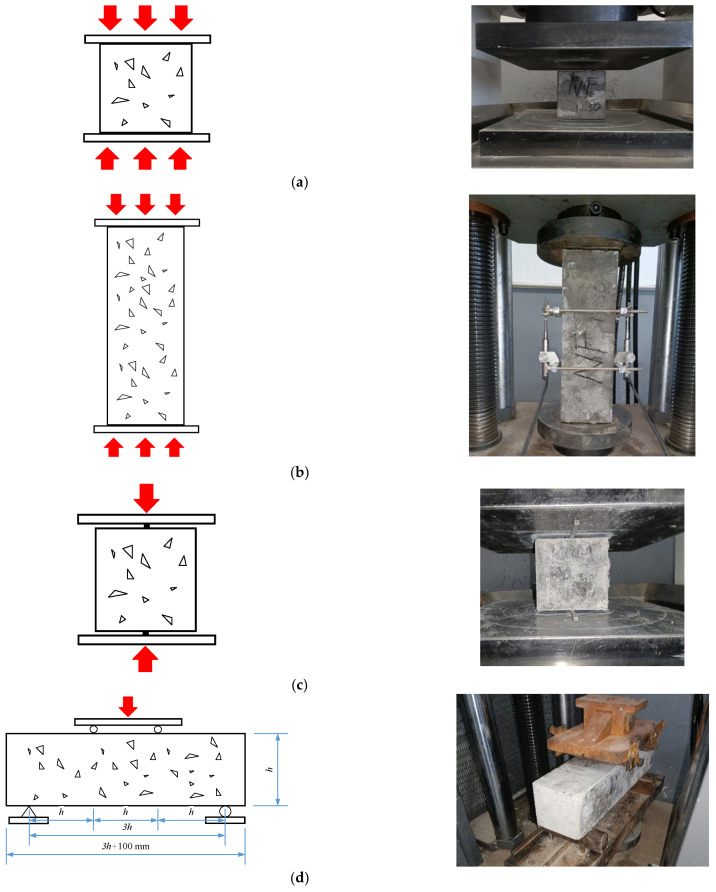
Mechanical performance tests: (**a**) cube compressive test; (**b**) axial compressive test; (**c**) splitting tensile test; (**d**) flexural test.

To investigate mechanical properties of SWSSC with POM fiber, the cube compressive, axial compressive, splitting tensile, and flexural tests were performed with reference to the code GB/T 50081-2002 [52], as shown in Figure 4. Considering the uncertainty of the mechanical properties of SWSSC, three specimens were prepared for each kind of mechanical property test and SWSSC mixture. For the cube and axial compressive tests, the loading rate was 0.5 MPa/s. The cube compressive strength (*f_cu_*) and axial compressive strength (*f_c_*) were determined through Equations (2) and (3), respectively, where *A* and *F* denote the bearing area and failure load of the test specimen, respectively. For the splitting tensile and flexural tests, the loading rate was 0.05 MPa/s. The splitting tensile strength (*f_t_*) and flexural strength (*f_f_*) were determined through Equations (4) and (5), respectively, where *A_s_*, *b*, and *h* denote the splitting area of the test specimen and the height and width of the cross-section, respectively, while *l* denotes the span between supports. Given the dimensions of the test specimens and the references in the code GB/T 50081-2002 [52], the conversion factors for *f_cu_*, *f_c_*, *f_t_*, and *f_f_* were 0.95, 0.95, 0.85, and 0.85, respectively.

(2) $$f_{cu}=\frac{F}{A}$$

(3) $$f_{c}=\frac{F}{A}$$

(4) $$f_{t}=\frac{2F}{\pi A_{s}}=0.637\frac{F}{A_{s}}$$

(5) $$f_{f}=\frac{Fl}{bh^{2}}$$

## 3. Results and Discussion

### 3.1. Effects of POM Fiber on the Workability of SWSSC

**Figure 5:**
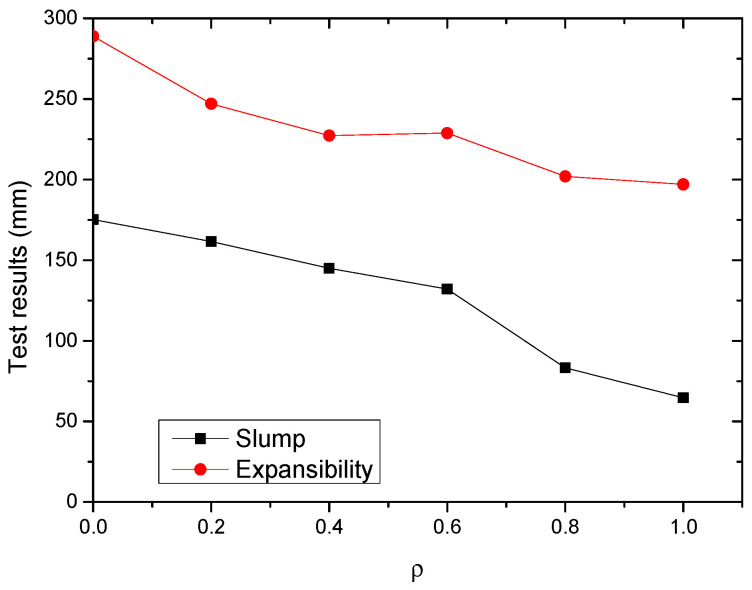
Test results of workability.

The test results of SWSSC with different *ρ* reflected the effects of POM fiber on workability. The slump and expansibility are shown in Figure 5 and Table 6. Based on the test results, with increases in *ρ*, the slump and expansibility decreased. Compared with the NF mixture, for the POM-1 mixture, the decreases in slump and expansibility were 110.6 and 91.9 mm, respectively. When *ρ* was larger than 0.6, there were obvious increases in the descent rates of slump and expansibility of the SWSSC mixtures.

### 3.2. Effects of POM Fiber on the Early-Age Cracking of SWSSC

Different indices are included in this section to quantify the early-age cracking performance of SWSSC mixtures with different *ρ*. The index *a_c_* determined by Equation (6) indicates the mean area per crack. In Equation (6), *N* denotes the number of cracks, *W_i_* denotes the peak width of crack *i*, and *L_i_* denotes the length of crack *i*. The index *b_c_* determined by Equation (7) indicates the number of cracks per unit area. In Equation (7), *A_s_* denotes the area of the slab. The index *c_c_* determined by Equation (8) indicates the total crack area per unit area, which was determined by the indices *a_c_* and *b_c_*. The coefficient of variation (COV) of the crack area was selected to quantify the uncertainty of the early-age cracking performance of SWSSC mixtures with different *ρ*.

(6) $$a_{c}=\frac{1}{2N}\sum_{i=1}^{N}\left(W_{i}\times L_{i}\right)$$

(7) $$b_{c}=N/A_{s}$$

(8) $$c_{c}=a_{c}\times b_{c}$$

**Figure 6:**
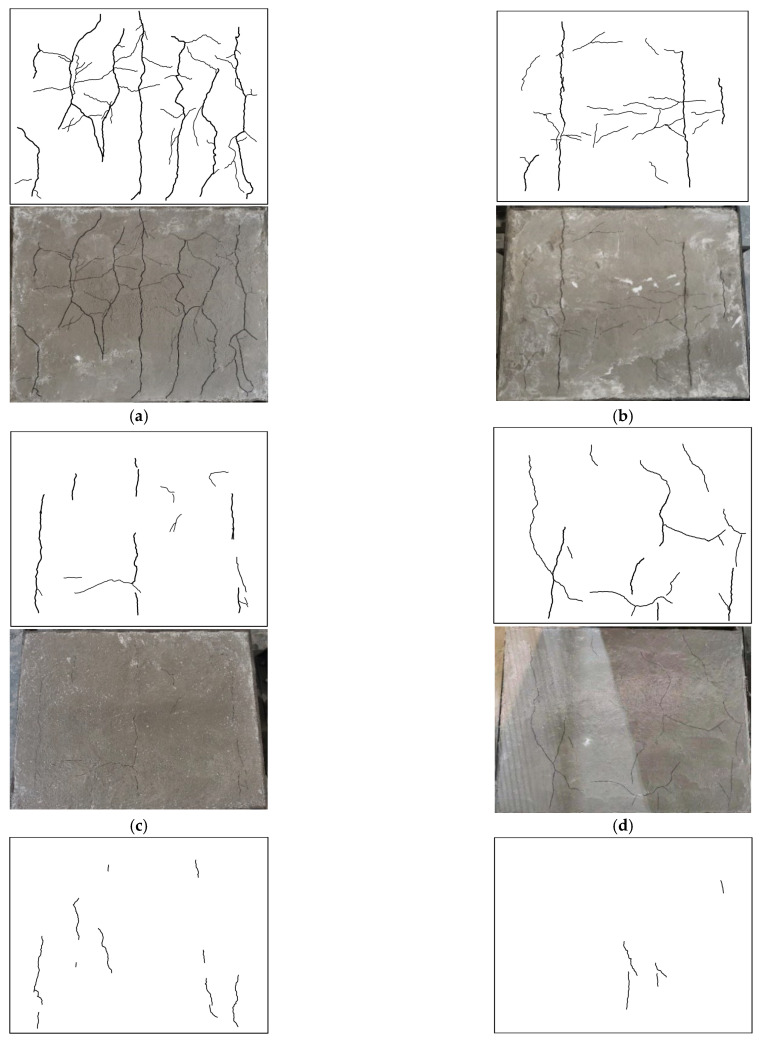

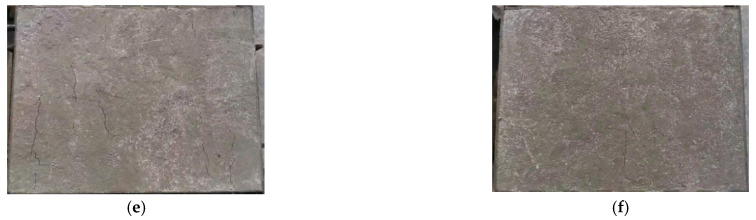
Early-age cracking performance: (**a**) POM-0; (**b**) POM-0.2; (**c**) POM-0.4; (**d**) POM-0.6; (**e**) POM-0.8; (**f**) POM-1.

**Figure 7:**
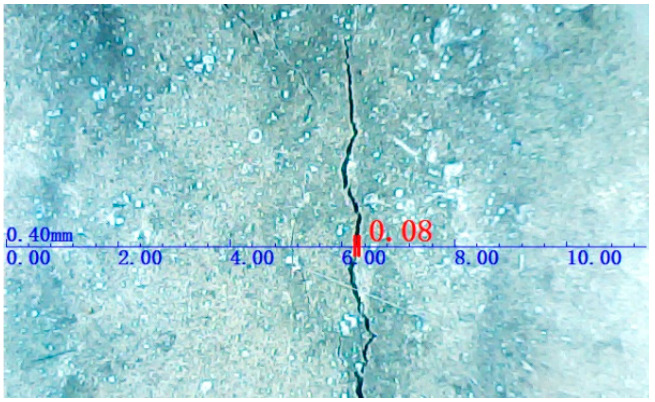
Method of measuring crack dimensions.

**Figure 8:**
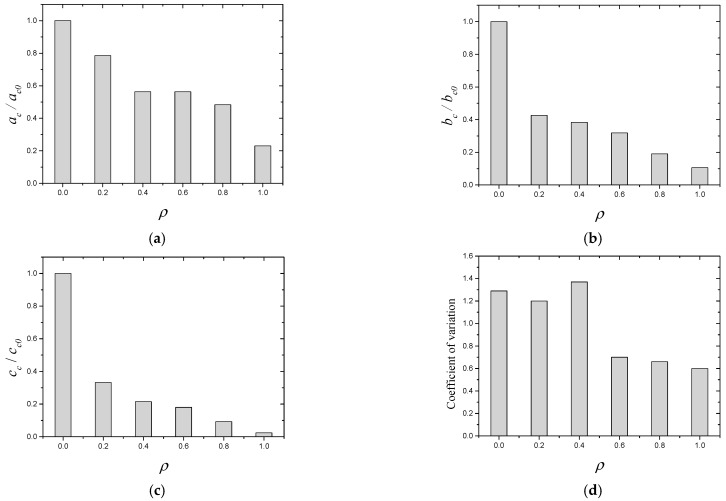
Early-age cracking performance of SWSSC mixtures with different *ρ*: (**a**) mean area per crack; (**b**) number of cracks per unit area; (**c**) total crack area per unit area; (**d**) coefficient of variation.

After the tests, the early-age crackling performances of all of the SWSSC specimens were recorded, as shown in Figure 6. It was clear that with increases in *ρ*, obvious increases in early-age cracking resistance were observed. A digital image processing technique was used to accurately determine the dimensions of the cracks, as shown in Figure 7. The measurement accuracy of the above technique was 0.01 mm. After calculation, the test results of early-age cracking performance were recorded, as listed in Table 7. It can be seen that with increases in *ρ*, the indices *a_c_*, *b_c_*_,_ and *c_c_* decreased. The descent rate of *c_c_* was greater than that of *a_c_* and *b_c_*, as shown in Figure 8. The POM-1 specimen showed the best early-age cracking resistance. Compared with those of the NF specimen, the *a_c_*, *b_c_*, and *c_c_* of the POM-1 specimen decreased by 77.0%, 89.4%, and 97.6%, respectively. When *ρ* was smaller than 0.4, the COV of the crack area was relatively large. When *ρ* was larger than 0.6, the COV of the crack area was relatively small. There was a clear decrease in the COV of the crack area when *ρ* increased from 0.4 to 0.6, as shown in Figure 8d. In general, the POM fiber had a significant effect on the improvement of the early-age cracking resistance of SWSSC.

### 3.3. Effects of POM Fiber on the Mechanical Performance of SWSSC

#### 3.3.1. Cube Compressive Performance

**Figure 9:**
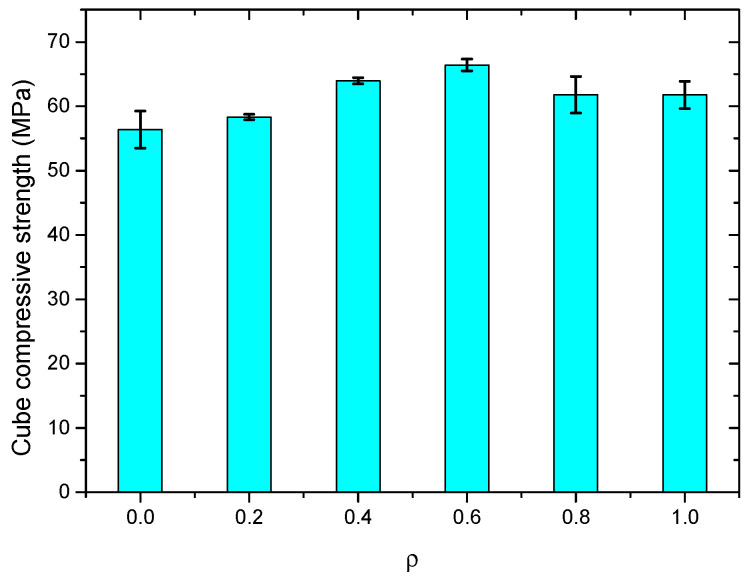
*f_cu_* of SWSSC specimens with different *ρ*.

**Figure 10:**
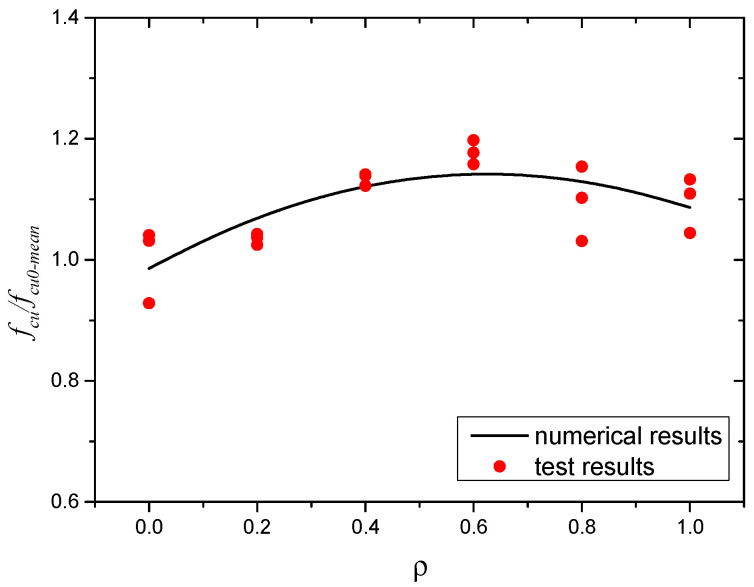
Comparison between numerical and test results: *f_cu_*.

**Figure 11:**
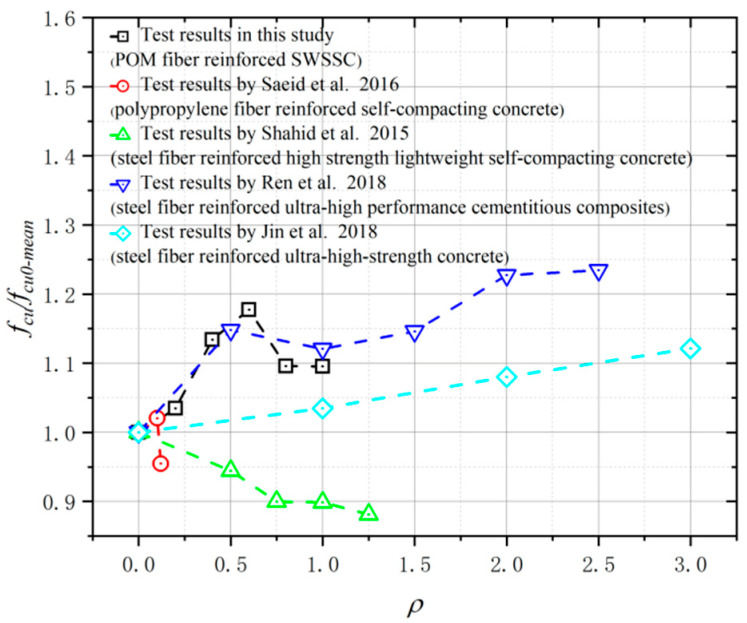
Comparison of compressive strength between POM-SWSSC and other types of fiber-reinforced concrete [58,59,60,61].

After testing, the results of cube compressive strength for all SWSSC specimens were obtained, as shown in Table 8. For each *ρ*, three specimens were included in the test. For the test results of all cube compressive specimens, the COV ranged from 0.0072 to 0.0510, indicating good stability. The mean value of three specimens was selected to determine the *f_cu_* of SWSSC with different *ρ*. It was clear that when *ρ* was smaller than 0.6%, with increases in *ρ*, the *f_cu_* of SWSSC increased, as shown in Figure 9. Under the compressive load, microcracks occurred in the concrete. With increases in the compressive load, microcracks developed and were connected to one another. Then, a failure surface was formed in the concrete. When there was POM fiber, transverse deformation of the concrete was constrained, which delayed the development of microcracks. Therefore, when the *ρ* was relatively small (0~0.6%), it had a beneficial effect on the *f_cu_*. Too many fibers in the SWSSC increased the difficulty of mixing the concrete, resulting in a decrease in the compactness of the SWSSC. The size and quantity of holes in the SWSSC increased, leading to the reduction in *f_cu_*. Therefore, based on test results, when *ρ* was larger than 0.6%, the increases in *ρ* led to decreases in *f_cu_*. Similar test results of fiber-reinforced concrete were observed in [48,53]. Based on the tests conducted in this study, the optimal value of *ρ* was 0.6%. For the *f_cu_* of SWSSC specimens with *ρ* = 0.6%, the *f_cu_* = 66.39 MPa, and the 95% confidence interval range was from 64.56 MPa to 68.22 MPa. To effectively quantify the effect of *ρ* on the *f_cu_* of SWSSC, a predictive equation was used in this study. Polynomial models are often used to fit experimental data [54,55,56,57]. A polynomial model was selected based on the cube compressive test results of SWSSC specimens with different *ρ*, as shown in Equation (9), where *ρ* is expressed in percentage terms. A comparison between the test and numerical results was performed, as shown in Figure 10, where *f_cu0-mean_* denotes the mean value of *f_cu*0*_*. It was believed that the proposed Equation (9) could be used to predict the *f_cu_* of SWSSC specimens with different *ρ* effectively. The mechanical properties of POM-SWSSC and other types of fiber-reinforced concrete were analyzed, as shown in Figure 11. It can be seen that the fiber reduced the compressive properties of the self-compacting concrete. However, for SWSSC and high-strength concrete, fiber could slightly improve their compressive strength, with an increase of about 10–20%. For POM-SWSSC, with the increase in fiber content (*ρ* ≤ 1%), the compressive strength was similar to that of steel-fiber-reinforced ultrahigh-performance cementitious composites, which first increased and then decreased.

**Figure 12:**
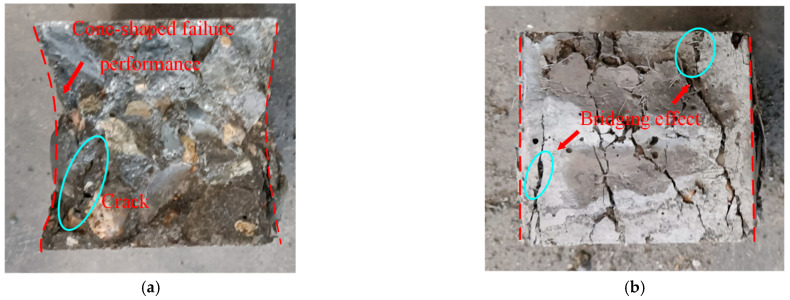
Failure performances of SWSSC specimens after the cube compressive test: (**a**) NF; (**b**) POM-0.6.

**Figure 13:**
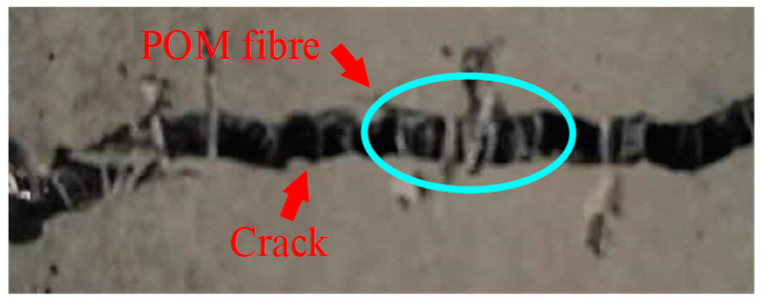
Bridging effect from POM fiber.

The effects of POM fiber on the failure performance of SWSSC specimens were observed (see Figure 12). The NF specimen was the control group without POM fiber. After the cube compressive test, the NF specimen was crushed. A typical cone-shaped failure performance was observed, as shown in Figure 12a. For SWSSC specimens with POM fiber, even though the spalling of concrete was observed, the degree was obviously less than that of the NF specimen. The POM fiber distributed between the vertical cracks exerted a bridging effect, as shown in Figure 13. For SWSSC specimens with POM fiber, there were connections between the cracks, unlike the NF specimen. The vertical cracks were restrained by the POM fiber. A similar phenomenon was observed in [53]. As a result, the integrity of specimens with POM fiber was almost entirely preserved. Furthermore, the stress concentration caused by the cracks in the concrete could be relieved by the POM fiber, which limited the development of cracks.

(9) $$f_{cu}=(-0.4017\rho^{2}+0.5023\rho +0.9857)f_{cu0-mean}$$

#### 3.3.2. Axial Compressive Performance

**Figure 14:**
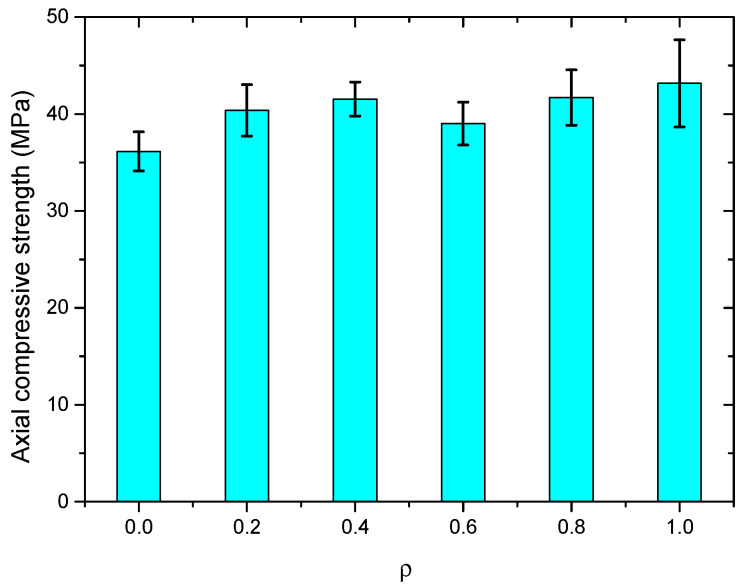
*f_c_* of SWSSC specimens with different *ρ*.

**Figure 15:**
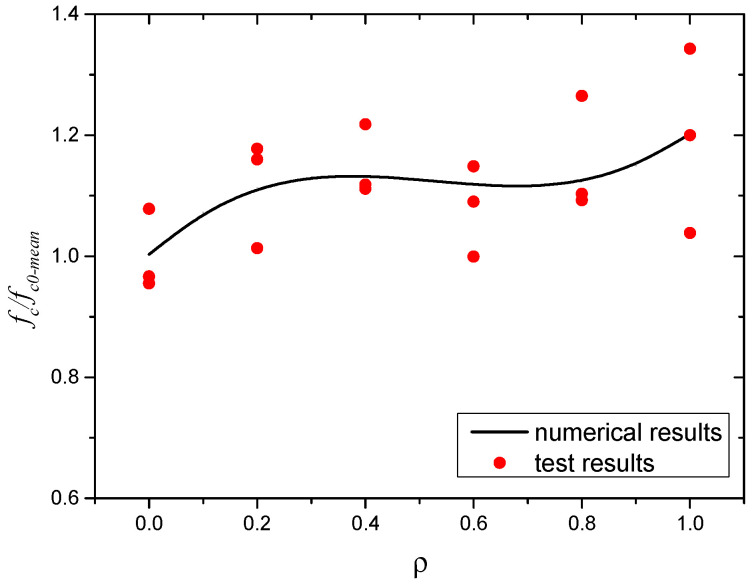
Comparison between numerical and test results: *f_c_*.

The test results of the axial compressive strength of the SWSSC specimens with different *ρ* are presented in Table 9. Compared with the test results of *f_cu_*, the uncertainty of the test results of *f_c_* was larger. The mean value of three specimens was selected to determine the *f_c_* of SWSSC. Based on the test results, when *ρ* was smaller than 0.4, with increases in *ρ*, the *f_c_* of the SWSSC specimens increased, as shown in Figure 14. When *ρ* = 0.6, there was a clear decrease in *f_c_*. For SWSSC specimens with *ρ* = 0.6~1.0, with increases in *ρ*, the *f_c_* increased. For all of the test results of axial compressive strength, the COV ranged from 0.0423 to 0.1042. The test results of SWSSC specimens with *ρ* = 1.0 showed the greatest uncertainty, with a standard deviation of 4.50 and COV of 0.1042. It is worth noting that the aforementioned uncertainty was also observed in many previous experimental studies on concrete [7,33,36,48]. For the SWSSC specimens with *ρ* = 1%, the *f_c_* = 43.16 MPa, and the 95% confidence interval range was from 34.16 MPa to 52.16 MPa. To quantify the effect of *ρ* on the *f_c_* of SWSSC, a predictive equation was used in this study. A polynomial was selected based on the axial compressive test results of SWSSC specimens with different *ρ*, as shown in Equation (10). A comparison between the test and numerical results was performed, as shown in Figure 15, where *f_c*0*-mean_* denotes the mean value of *f_*c0*_*. It was believed that the proposed Equation (10) could be used to predict the change trend of *f_c_* of SWSSC specimens with different *ρ*. 

**Figure 16:**
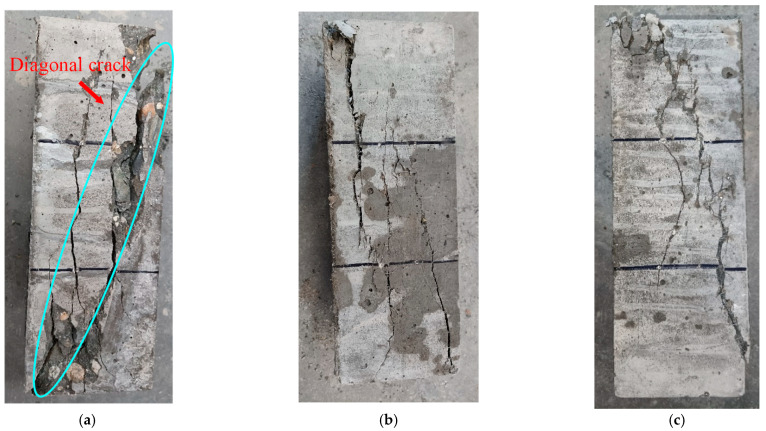

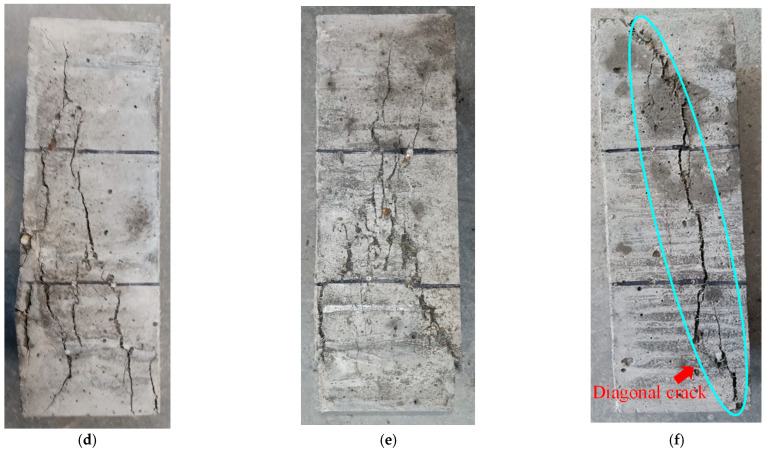
Failure performances of SWSSC specimens after the axial compressive test: (**a**) NF; (**b**) POM-0.2; (**c**) POM-0.4; (**d**) POM-0.6; (**e**) POM-0.8; (**f**) POM-1.

After the axial compressive test, the failure performances of SWSSC specimens with different *ρ* were recorded, as shown in Figure 16. For the NF specimen, the vertical microcracks occurred in the test specimen at the beginning of loading. With increases in the loading procedure, the microcracks merged into diagonal cracks. Then, spalling of the concrete was observed, as shown in Figure 16a. For SWSSC specimens with POM fiber, there were connections between the cracked concretes. Even though the spalling of concrete was observed, it was effectively limited by the POM fiber in the SWSSC. The above failure performance of the axial compressive test specimens was similar to that of cube compressive test specimens. Because of the aforementioned bridging effect, the microcracks were restrained by the POM fiber. The transition from microcracks to macrocracks was thereby delayed. The integrity of the SWSSC specimens with POM fiber was almost entirely preserved—especially for SWSSC specimens with *ρ* = 0.4%, 0.6%, and 0.8%. The cracking features of the POM-0.8 specimen were clearly different from those of the NF specimen. Compared with the NF specimen, the cracks in the POM-0.8 specimen were dispersed across a larger area. The reason for this might be that the POM fiber in the SWSSC alleviated the stress concentration at the crack location, enhancing the ductility of the SWSSC specimen. As for the POM-1 specimen, the presence of too many POM fibers in the SWSSC reduced the compactness. There was a diagonal crack in the POM-1 specimen, which was similar to that of the NF specimen. In general, the ductility of the axial compressive test specimens was improved by the POM fiber in the SWSSC.

(10) $$f_{c}=(1.149\rho^{3}-1.820\rho^{2}+0.8701\rho +1.003)f_{c0-mean}$$

#### 3.3.3. Splitting Tensile Performance

**Figure 17:**
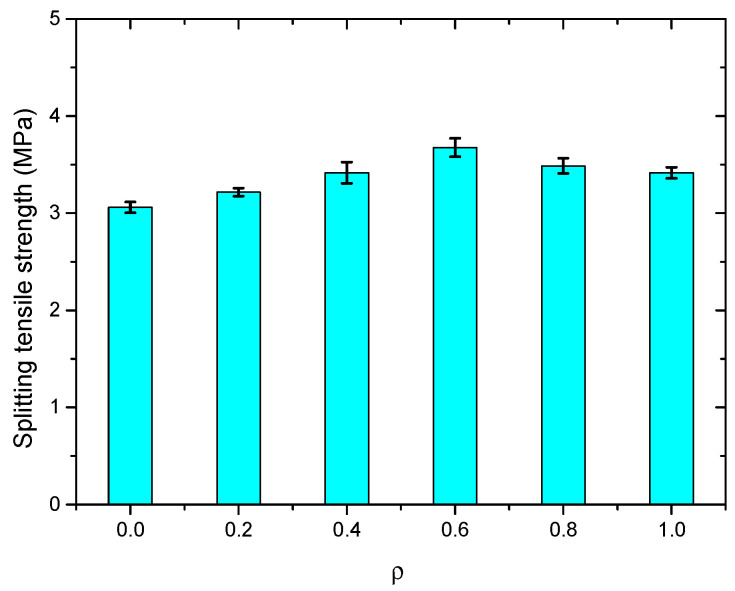
*f_t_* of SWSSC specimens with different *ρ*.

**Figure 18:**
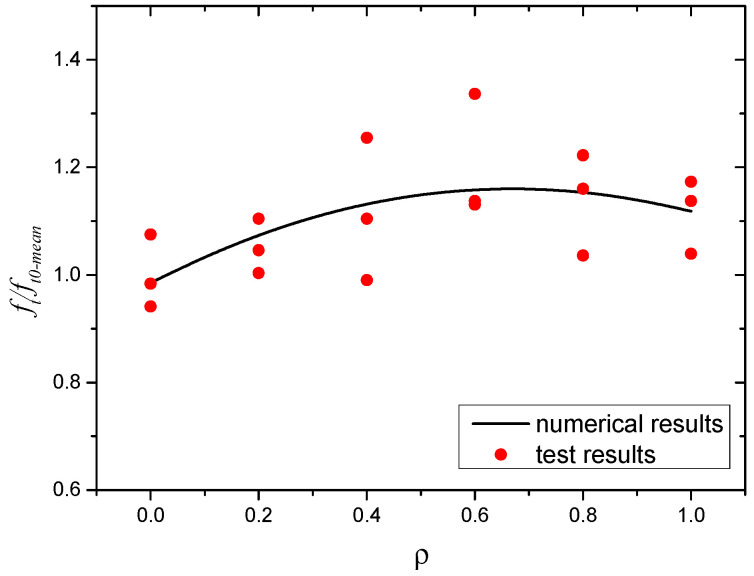
Comparison between numerical and test results: *f_t_*.

**Figure 19:**
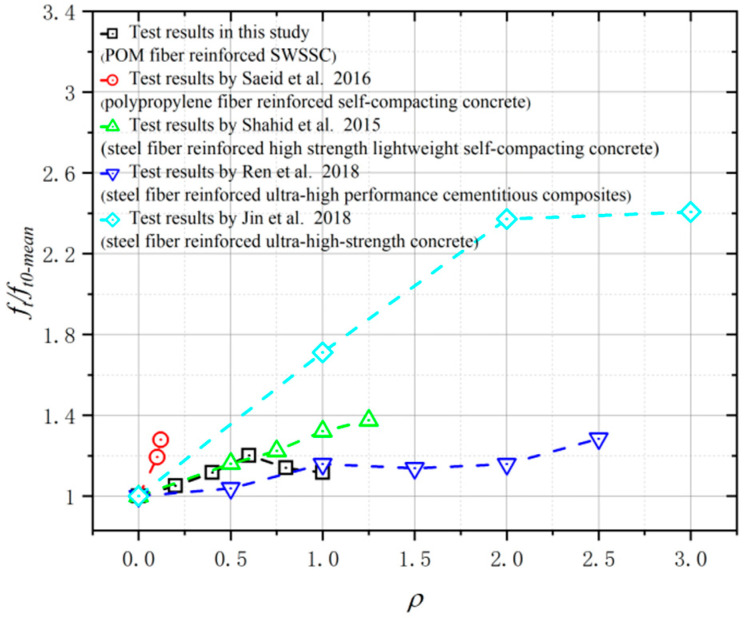
Comparison of tensile strength between POM-SWSSC and other types of fiber-reinforced concrete [58,59,60,61].

The splitting tensile test results of the SWSSC specimens with different *ρ* were recorded, as shown in Table 10. For all of the test results of splitting tensile strength, the COV ranged from 0.0395 to 0.0971. The mean value of three specimens was selected to determine the *f_t_* of SWSSC specimens with different *ρ*. It was clear that with increases in the *ρ*, the *f_t_* of SWSSC specimens with different *ρ* exhibited unimodal distribution, as shown in Figure 17. Hence, there was an optimal value of *ρ*. When *ρ* was smaller than 0.6, with increases in *ρ*, the *f_t_* of the SWSSC specimens increased. As mentioned previously, the presence of too many POM fibers led to increases in the size and quantity of holes in the concrete, reducing its mechanical properties. Then, when *ρ* was larger than 0.6, the increases in *ρ* led to decreases in *f_t_*. Compared with that of the NF specimen, the *f_t_* of the POM-0.2, POM-0.4, POM-0.6, POM-0.8, and POM-1 specimens increased by 5.1%, 11.6%, 20.2%, 13.9%, and 11.6%, respectively. Based on the tests conducted in this study, the optimal value of *ρ* was 0.6%. For the SWSSC specimens with *ρ* = 0.6%, the *f_t_* = 3.68 MPa, and the 95% confidence interval range was from 3.09 MPa to 4.26 MPa. A predictive equation was determined to effectively clarify the effect of *ρ* on the *f_t_* of the SWSSC specimens. Given the distribution features of the test results, a polynomial was proposed based on the splitting tensile test results of the SWSSC specimens with different *ρ*, as shown in Equation (11). A comparison between the test and numerical results was performed, as shown in Figure 18, where *f_t0-mean_* denotes the mean value of *f_t0_*. Therefore, the proposed Equation (11) could be used to effectively predict the *f_t_* of SWSSC specimens with different *ρ*. The tensile strength of POM-SWSSC and other types of fiber-reinforced concrete was analyzed, as shown in Figure 19. Unlike the compressive properties (Figure 11), the fiber could improve the tensile strength of concrete—especially for steel-fiber-reinforced ultra-high-strength concrete. With the addition of fiber, the tensile strength of the ultrahigh-strength concrete was improved by more than 120%. The tensile strength of POM-SWSSC was improved by about 20%, which is slightly lower than of the increase seen in other fiber-reinforced concretes.

**Figure 20:**
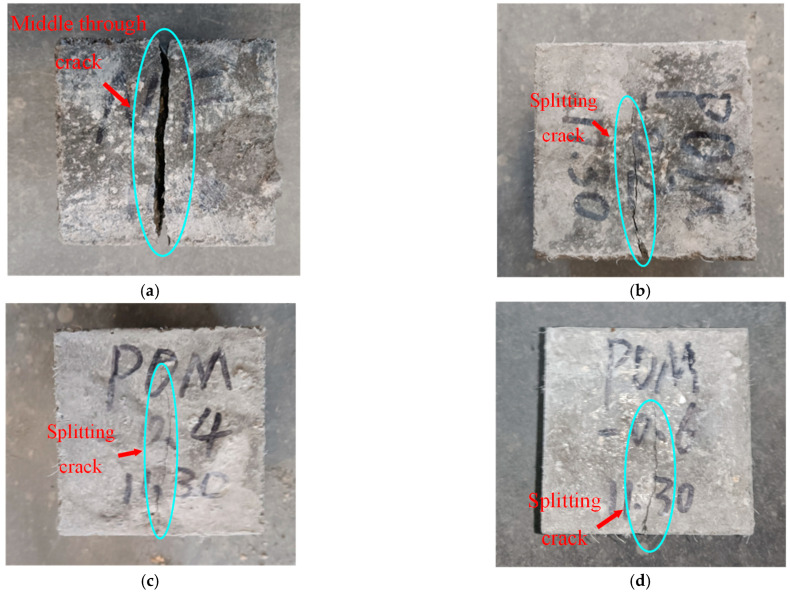

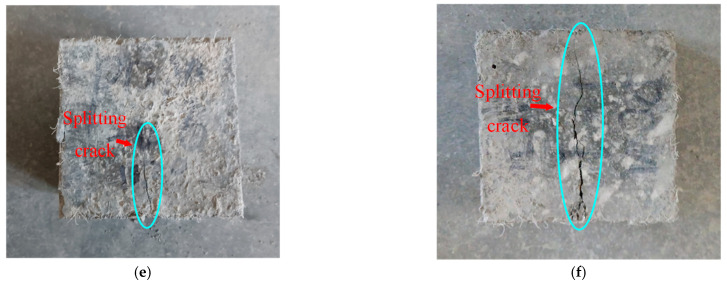
Failure performance of splitting tensile tests: (**a**) NF; (**b**) POM-0.2; (**c**) POM-0.4; (**d**) POM-0.6; (**e**) POM-0.8; (**f**) POM-1.

**Figure 21:**
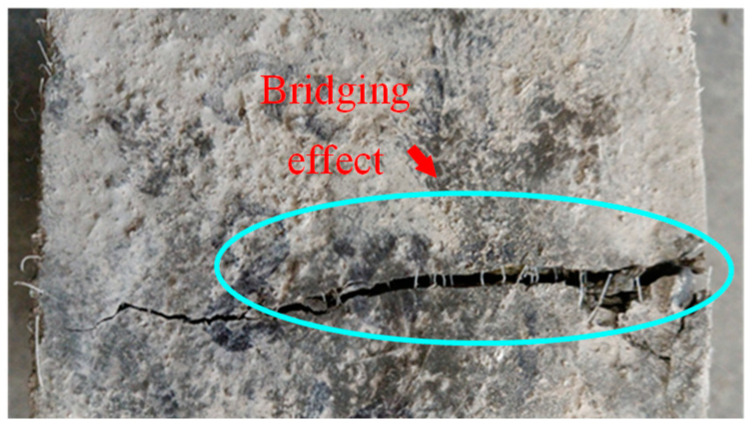
Bridging effect in the POM-0.8-3 splitting tensile test specimen.

The failure performances of the splitting tensile test specimens were observed, as shown in Figure 20. For the NF specimen, cracks occurred in the middle of the SWSSC specimens with increases in load. A clear separation in concretes was observed, indicating a brittle failure mode. For the SWSSC specimens with different *ρ*, the cracks were restrained by the POM fiber. The crack widths of the POM-SWSSC specimens were much smaller than those of the NF specimen, as shown in Figure 20. The bridging effect caused by the POM fiber was observed in the cracks, as shown in Figure 21. No separation was observed in the POM-SWSSC specimens. After the splitting tensile test, the integrity of the SWSSC specimens with POM fiber was preserved. The stress concentration caused by the cracks was relieved by the POM fiber, which delayed the crack propagation. The SWSSC specimens with POM fiber tended to exhibit a ductile failure mode. The cracks in the POM-1 specimen were more obvious than those in other test specimens with POM fiber, which might be because the presence of too many fibers in the SWSSC reduced its compactness. To obtain the best enhancing effect of POM fiber on the splitting tensile performance of SWSSC, it is important to determine the proper *ρ*.

(11) $$f_{t}=(-0.3929\rho^{2}+0.5261\rho +0.9852)f_{t0-mean}$$

#### 3.3.4. Flexural Performance

**Figure 22:**
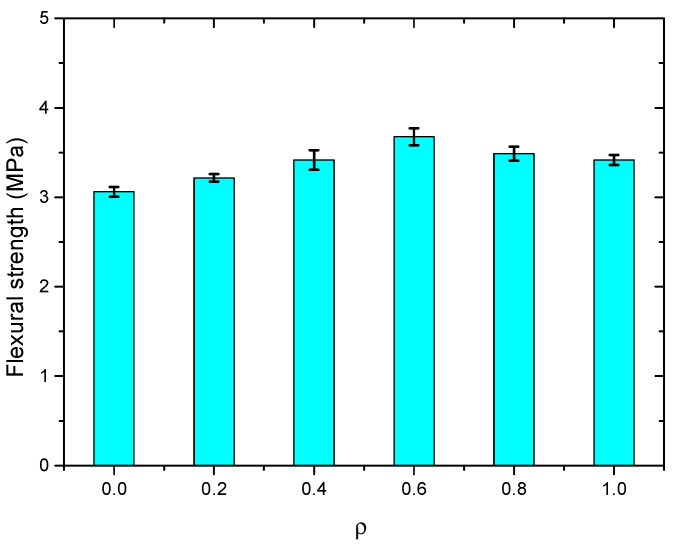
*f_f_* of SWSSC specimens with different *ρ*.

**Figure 23:**
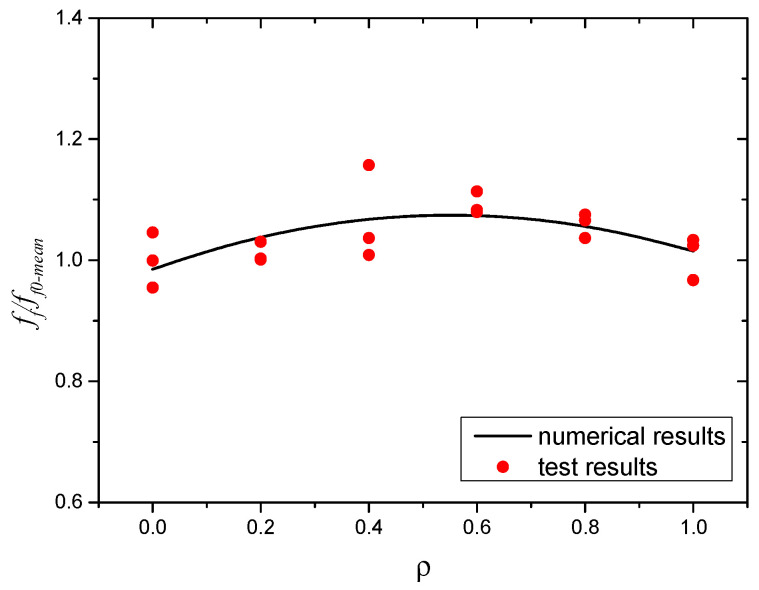
Comparison between numerical and test results: *f_f_*.

The flexural test results of the SWSSC specimens are shown in Table 11. For all of the flexural specimens, the COV ranged from 0.0140 to 0.0605, showing good stability. The mean value of three specimens was selected to determine the *f_f_* of the SWSSC specimens with different *ρ*. It can be seen that when the *ρ* was smaller than 0.6, with increases in *ρ*, the *f_f_* of the SWSSC specimens increased, as shown in Figure 22. However, when the *ρ* was larger than 0.6, the increase in *ρ* resulted in decreases in *f_f_*, similar to the *f_t_* of the SWSSC specimens. Based on the test results, the optimal value of *ρ* was 0.6%. For the test results of SWSSC specimens with *ρ* = 0.6%, the *f_f_* = 7.08 MPa, and the 95% confidence interval range was from 6.88 MPa to 7.28 MPa. Compared with that of NF specimens, the *f_f_* of the POM-0.2, POM-0.4, POM-0.6, POM-0.8, and POM-1 specimens were increased by 1.1%, 6.7%, 9.2%, 5.9%, and 0.8%, respectively. A predictive equation was proposed to effectively quantify the effect of *ρ* on the *f_f_* of the SWSSC specimens. A polynomial was selected based on the flexural test results of SWSSC specimens with different *ρ*, as shown in Equation (12). A comparison between the test and numerical results was performed, as shown in Figure 23, where *f_f*0*-mean_* denotes the mean value of *f_f*0*_*. It was believed that the proposed Equation (12) could be used to effectively predict the *f_f_* of SWSSC specimens with different *ρ*.

**Figure 24:**
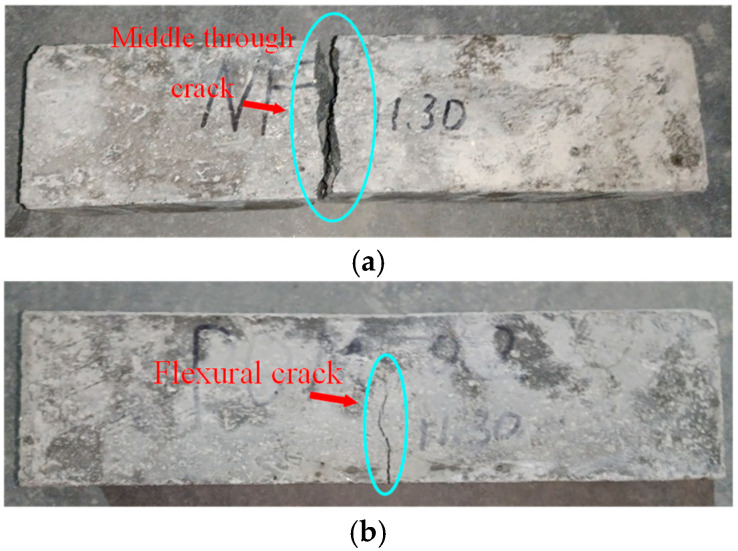

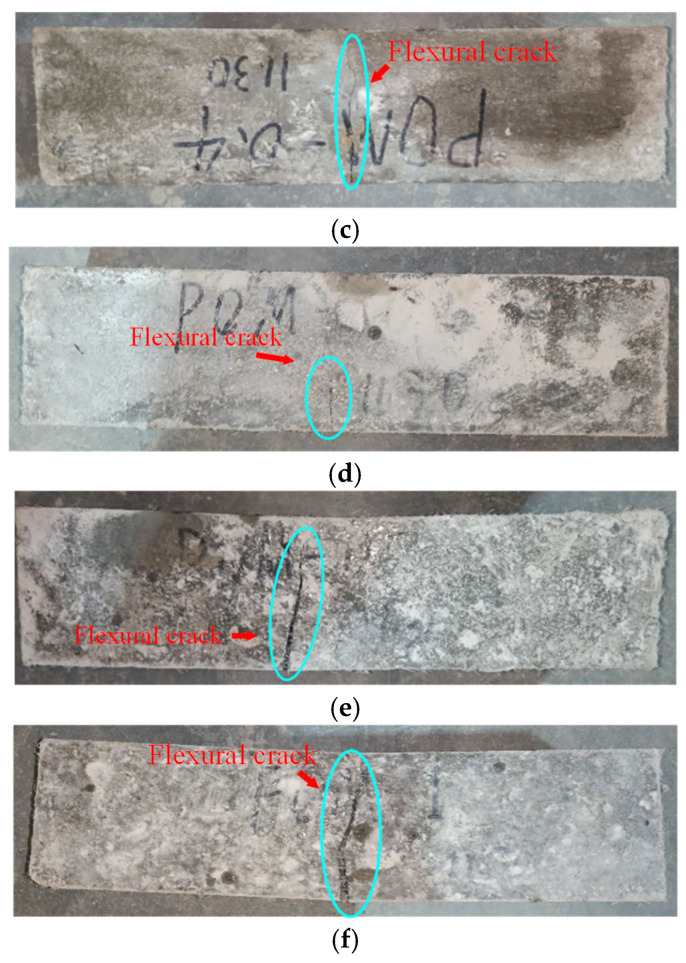
Failure performance of flexural tests: (**a**) NF; (**b**) POM-0.2; (**c**) POM-0.4; (**d**) POM-0.6; (**e**) POM-0.8; (**f**) POM-1.

After the flexural tests, the failure performances of the SWSSC specimens were recorded, as shown in Figure 24. For the NF specimen, cracks in the concrete developed quickly. Then, the NF specimen suddenly broke into two pieces. There was no advanced phenomenon observed before the failure. Therefore, the NF specimen exhibited a brittle failure mode after the flexural test. As for the test specimens with POM fiber, cracking was restrained by the POM fiber. The crack widths of the test specimens with POM fiber were much smaller than those of the NF specimen. Even though cracks occurred in the flexural test specimens with POM fiber, no separation was observed, indicating that the ductility of the SWSSC specimens was enhanced by POM fiber. Because of the bridging effect caused by the POM fiber, the integrity of the SWSSC specimens with POM fiber was almost entirely preserved after the flexural test. When *ρ* = 0.6, the POM fiber showed the most significant effects in reducing the length and width of flexural cracks (see Figure 24).

(12) $$f_{f}=(-0.2977\rho^{2}+0.3276\rho +0.9851)f_{f0-mean}$$

### 3.4. Complete Stress–Strain Curve of SWSSC

**Figure 25:**
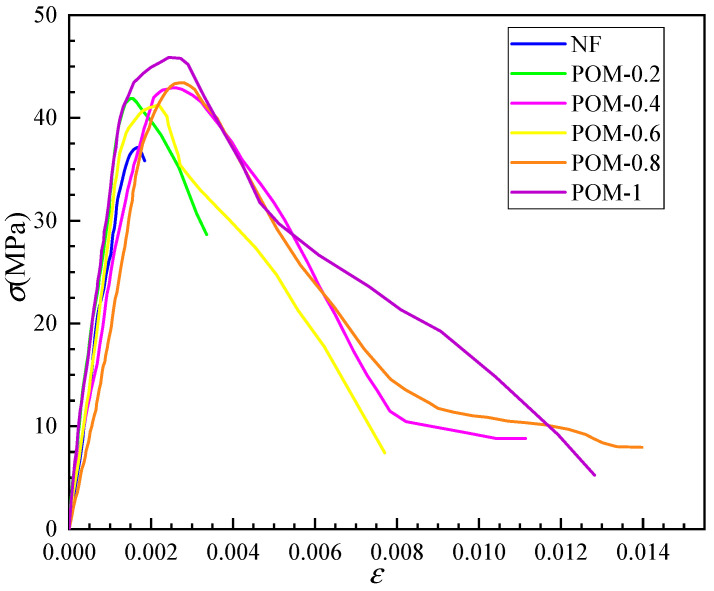
Complete stress–strain curve under uniaxial compression.

**Figure 26:**
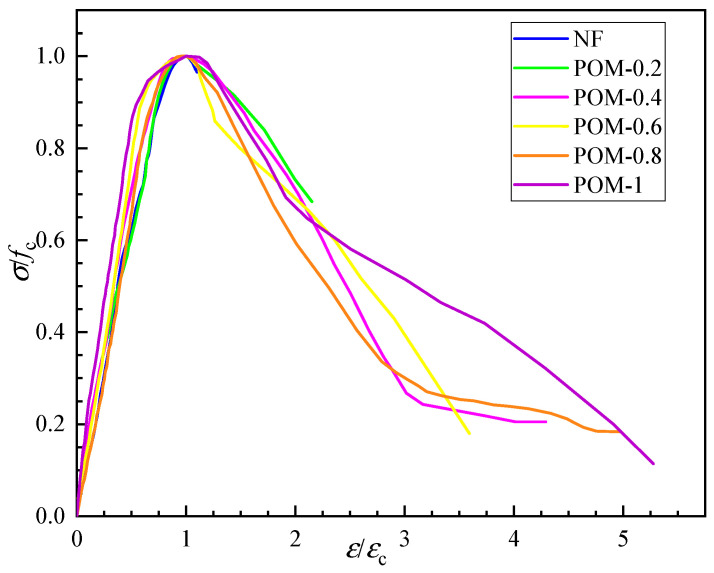
Dimensionless complete stress–strain curve.

The complete stress–strain curve of SWSSC was obtained according to the axial compressive test (Figure 25). To better describe the stress–strain evolution law of SWSSC with different *ρ*, the dimensionless stress and strain data were used to draw dimensionless complete stress–strain curves, as shown in Figure 26. Due to the brittle failure of SWSSC without fiber, the specimens failed instantaneously when the peak load was reached. As a result, the stress and strain data after the peak point could not be obtained, so the complete stress–strain curve of NF had only an ascending segment. The POM fiber could help to prevent the development of cracks in the concrete, slowing down the failure process [35]. Therefore, complete stress and strain data of POM-0.2–POM-1 could be obtained. 

The axial compression process of SWSSC can be divided into four stages: When *σ* < 0.3*f_c_*, cracks had not yet initiated, and there was mainly elastic deformation in the specimen. Therefore, the anti-cracking effect of POM fiber could not be exerted. The stress–strain curves of SWSSC with and without POM fiber were essentially the same, showing a linear upward trend. As the load increased (0.3 *f_c_* < *σ* <0.85 *f_c_*), a large number of microcracks appeared on the surface of the specimen and extended from the loading end to the subsurface. At this stage, the cracks developed stably, and there were no obvious macroscopic cracks on the surface of the specimen. The stress–strain curve was convex and nonlinear. When 0.85 *f_c_* < *σ* < *f_c_*, macroscopic cracks began to appear on the surface of the specimen and developed rapidly due to the acceleration of horizontal expansion. The crack propagation rate of POM-SWSSC was reduced to a certain extent due to the anti-cracking effect of the POM fiber. When the load reached the peak value, the cracks penetrated and formed a failure surface. There were obvious macroscopic cracks on the surface, and the fibers passing through the failure surface were pulled out or broken from the matrix. The stress–strain curves of the POM-SWSSC specimens at this stage became gentler compared with that of NF-SWSSC. Furthermore, the gentle degree of the stress–strain curve was positively correlated with the *ρ*. The specimens showed better toughness and ductility with the increase in *ρ*. After the stress reached *f_c_*, the bearing capacity decreased rapidly, and the stress–strain curve dropped suddenly. The NF-SWSSC specimens were rapidly destroyed at this stage, so the complete descent stage data could not be obtained. Meanwhile, the descending sections of the stress–strain curves of the POM-SWSSC specimens were complete, because the fibers that were not pulled out or broken bore part of the load, delaying the failure process of the specimen.

**Figure 27:**
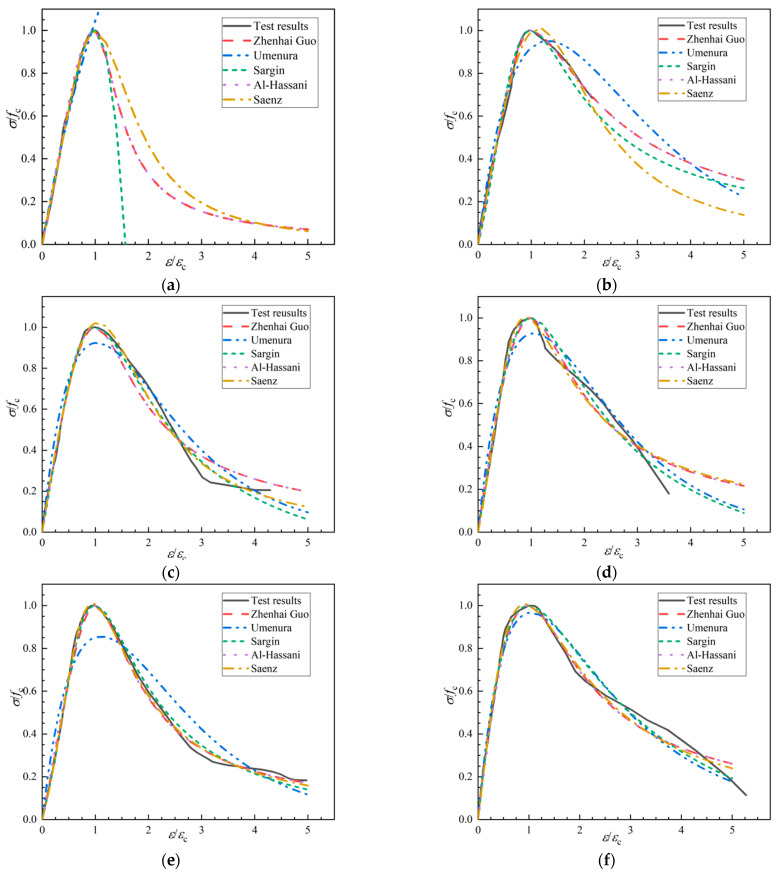
Fitting results of complete stress–strain curves of each group of concrete under axial compression: (**a**) NF; (**b**) POM-0.2; (**c**) POM-0.4; (**d**) POM-0.6; (**e**) POM-0.8; (**f**) POM-1.

In order to better describe the stress–strain relationship of concrete, we proposed a variety of typical equations for the complete stress–strain curve of concrete under uniaxial compression (Equations (13)–(17)). The rising section of the stress–strain curve was generally described by polynomial, exponent, and rational fraction equations, while the descending section was described by rational fraction equations (Table 12). Since the shape of the complete stress–strain curve of SWSSC was close to that of ordinary concrete, the above typical models were used to describe the stress–strain relationship of SWSSC. Based on the test data, the stress–strain relationship of SWSSC with and without POM was fitted with the above typical models. The fitting results and fitting accuracy are given in Table 13. As shown in Figure 27, it was found that except for the exponent equations, the fitting results of the other types of equations were in good agreement with the test results, with R^2^ > 0.98.

The polynomial model proposed by Guo Zhenhai [62] has only two coefficients, making it simpler than other models. According to the test results, it was found that this model was accurate in describing the stress–strain relationships of SWSSC with and without POM fiber. Therefore, this polynomial model was suggested to quantify the stress–strain relationships of SWSSC with and without POM fiber. The fiber volume fractions (ρ) affected the stress–strain relationship of the SWSSC. Based on the test results, the influence of the fiber volume fraction (ρ) on the stress–strain model was analyzed. Equations (18) and (19) describe the relationships between the model coefficients (A and B, respectively) and ρ. According to Equations (13), (18), and (19), the complete stress–strain curve of SWSSC with any POM fiber content could be determined.

(18) $$A=42.47\rho^{4}-77.35\rho^{3}+41.27\rho^{2}-5.066\rho +1.091\;\;\;R^{2}=0.9687$$

(19) $$B=39.82\rho^{4}-104.2\rho^{3}+91.56\rho^{2}-30.38\rho +4.079\;\;\;R^{2}=0.9632$$

## 4. Conclusions

In this study, POM fiber was innovatively applied to SWSSC, and the fresh and hardened properties of SWSSC reinforced with POM fiber were experimentally studied. The effects of the POM fiber on the workability, early-age cracking performance, and mechanical properties of SWSSC with different *ρ* were discussed based on the experimental results. The main conclusions are as follows:

(1) The workability of SWSSC mixtures with different *ρ* was investigated through the experiment. With increases in *ρ*, the slump and expansibility decreased. Compared with the NF mixture, for the POM-1 mixture, the decreases in slump and expansibility were 110.6 and 91.9 mm, respectively. When *ρ* was larger than 0.6, there were obvious increases in the descent rates of slump and expansibility of the SWSSC mixtures.
(2) Based on the knife-edge binding method, experiments on the early-age cracking performance of SWSSC mixtures with different *ρ* were conducted. The test results indicated that the POM fiber had a significant effect in improving the early-age cracking resistance of SWSSC. Compared with those of the NF specimens, the *a_c_*, *b_c_*, and *c_c_* of the POM-1 specimens decreased by 77.0%, 89.4%, and 97.6%, respectively.
(3) Cube and axial compressive tests were conducted to clarify the effects of POM fiber on the compressive properties of SWSSC. When *ρ* was smaller than 0.6, with increases in *ρ*, the *f_cu_* of the SWSSC specimens increased. When *ρ* was larger than 0.6, the increase in *ρ* led to decreases in *f_cu_*. When *ρ* was smaller than 0.4, with increases in *ρ*, the *f_c_* of the SWSSC specimens increased. When *ρ* = 0.6, there was a clear decrease in *f_c_*. For SWSSC specimens with *ρ* = 0.6~1.0, with increases in *ρ*, the *f_c_* increased.
(4) The *f_t_* and *f_f_* of SWSSC specimens with different *ρ* exhibited unimodal distribution. Compared with that of the NF specimen, the *f_t_* of the POM-0.2, POM-0.4, POM-0.6, POM-0.8, and POM-1 specimens was increased by 5.1%, 11.6%, 20.2%, 13.9%, and 11.6%, respectively. Results of flexural test indicated that compared with that of the NF specimens, *f_f_* of POM-0.2, POM-0.4, POM-0.6, POM-0.8 and POM-1 specimens were increased by 1.1%, 6.7%, 9.2%, 5.9% and 0.8%, respectively.
(5) The failure performances of the test specimens after cube and axial compressive tests, splitting tensile tests, and flexural tests were recorded. For the NF specimens, brittle failure modes were observed. For the test specimens with POM fiber, the stress concentration caused by cracks in the concrete was relieved by the POM fiber. POM fiber performed a bridging effect, building connections between cracked concretes. The integrity of the SWSSC specimens with POM fiber was almost entirely preserved, indicating ductile failure modes.
(6) To quantify the effects of POM fiber on the *f_cu_*, *f_c_*, *f_t_*, and *f_f_* of SWSSC with different *ρ*, predictive equations were proposed based on the test results. Comparisons between the results from predictive equations and the test results were performed, proving the effectiveness of the predictive equations.
(7) The effects of different stress–strain models in describing the stress–strain relationships of SWSSC with and without POM fiber were compared, and a polynomial model suitable for SWSSC was suggested. According to the model, the complete stress–strain curve of SWSSC with any POM fiber content could be determined.

## Figures and Tables

**Table 1:** Chemical composition of tap water, seawater, and sea sand.

	Unit	Cl^−^	SO_4_^2−^	Na^+^	K^+^	Mg^2+^	Ca^2+^
Tap water	mg/L	12.3	36.8	8.7	2.8	9.6	53.1
Seawater	mg/L	19,365.5	2537.5	11,208.7	389.9	1321.7	395.8
Sea sand	mg/kg	7.4	34.9	14.2	4.0	3.0	13.8

**Table 2:** Screening test results of sea sand.

	Sieve Diameter	Cumulative Percentages of Sieve Residue (%)
*A*_1_	4.75 mm	4.0
*A*_2_	2.36 mm	14.0
*A*_3_	1.18 mm	29.4
*A*_4_	600 μm	49.0
*A*_5_	300 μm	68.6
*A*_6_	150 μm	90.6
	<150 μm	99.4

**Table 3:** Physical properties of the POM fiber.

Fiber	Density (kg/m^3^)	Tensile Strength (MPa)	Elongation (%)	Melting Point (°C)	Elastic Modulus (GPa)
POM	1400	970	18	165	8

**Table 4:** Proportions of all SWSSC mixtures.

Types	Cement (kg/m^3^)	Fly Ash (kg/m^3^)	Mineral Powder (kg/m^3^)	Sea Sand (kg/m^3^)	Coarse Aggregate (kg/m^3^)	Seawater (kg/m^3^)	Fiber (kg/m^3^)	W/C Ratio
NF	264	88	88	831	1016	160	0.0	0.36
POM-0.2	264	88	88	831	1016	160	2.8	
POM-0.4	264	88	88	831	1016	160	5.6	
POM-0.6	264	88	88	831	1016	160	8.4	
POM-0.8	264	88	88	831	1016	160	11.2	
POM-1	264	88	88	831	1016	160	14.0	

**Table 5:** Details of specimens.

Test Type	Specimen Size	Quantity of Specimens					
		NF	POM-0.2	POM-0.4	POM-0.6	POM-0.8	POM-1
Workability test	-	1	1	1	1	1	1
Early-age cracking test	800 mm × 600 mm × 100 mm	1	1	1	1	1	1
Cube compressive test	100 mm × 100 mm × 100 mm	3	3	3	3	3	3
Axial compressive test	100 mm × 100 mm × 300 mm	3	3	3	3	3	3
Splitting tensile test	100 mm × 100 mm × 100 mm	3	3	3	3	3	3
Flexural test	100 mm × 100 mm × 400 mm	3	3	3	3	3	3

**Table 6:** Test results of the workability of SWSSC.

Types	Slump (mm)	Expansibility 1 (mm)	Expansibility 2 (mm)	Expansibility (mm)
NF	175.2	291.3	286.5	288.9
POM-0.2	161.5	244.3	249.6	247.0
POM-0.4	144.9	231.2	223.4	227.3
POM-0.6	132.1	235.1	222.5	228.8
POM-0.8	83.2	204.4	199.3	201.9
POM-1	64.6	198.1	195.8	197.0

**Table 7:** Test results of early-age cracking performance.

Number	*a_c_* (mm^2^)	*b_c_* (m^−2^)	*c_c_* (mm^2^/m^2^)	COV
NF	12.6	97.9	1237.9	1.29
POM-0.2	9.9	41.7	411.1	1.20
POM-0.4	7.1	37.5	266.4	1.37
POM-0.6	7.1	31.2	222.7	0.70
POM-0.8	6.1	18.7	114.3	0.66
POM-1	2.9	10.4	30.2	0.60

**Table 8:** Results of compressive tests of SWSSC with different mixes.

Number	*f_cu_* (MPa)	*f_cu-mean_* (MPa)	Standard Deviation	COV	95% Confidence Interval	
					Lower Range	Upper Range
NF-1	58.67	56.38	2.87	0.0510		
NF-2	58.15				50.64	62.13
NF-3	52.33					
POM-0.2-1	58.78	58.33	0.42	0.0072		
POM-0.2-2	58.45				57.49	59.17
POM-0.2-3	57.77					
POM-0.4-1	63.28	63.95	0.48	0.0075		
POM-0.4-2	64.23				63.00	64.91
POM-0.4-3	64.35					
POM-0.6-1	65.29	66.39	0.91	0.0138		
POM-0.6-2	66.36				64.56	68.22
POM-0.6-3	67.53					
POM-0.8-1	62.15	61.78	2.85	0.0461		
POM-0.8-2	58.12				56.08	67.48
POM-0.8-3	65.07					
POM-1-1	62.55	61.77	2.11	0.0342		
POM-1-2	63.88				57.54	66.00
POM-1-3	58.88					

**Table 9:** Results of the axial compressive tests of SWSSC with different mixes.

Number	*f_c_* (MPa)	*f_c-mean_* (MPa)	Standard Deviation	COV	95% Confidence Interval	
					Lower Range	Upper Range
NF-1	34.94	36.15	2.01	0.0555		
NF-2	34.53				32.13	40.17
NF-3	38.98					
POM-0.2-1	41.94	40.38	2.66	0.0660		
POM-0.2-2	42.57				35.05	45.71
POM-0.2-3	36.63					
POM-0.4-1	40.43	41.55	1.76	0.0423		
POM-0.4-2	40.18				38.03	45.06
POM-0.4-3	44.03					
POM-0.6-1	39.41	39.02	2.22	0.0568		
POM-0.6-2	41.52				34.58	43.46
POM-0.6-3	36.13					
POM-0.8-1	45.73	41.70	2.85	0.0684		
POM-0.8-2	39.88				36.00	47.41
POM-0.8-3	39.50					
POM-1-1	48.55	43.16	4.50	0.1042		
POM-1-2	43.38				34.16	52.16
POM-1-3	37.54					

**Table 10:** Results of the splitting tensile tests of SWSSC with different mixes.

Number	*f_t_* (MPa)	*f_t-mean_* (MPa)	Standard Deviation	COV	95% Confidence Interval	
					Lower Range	Upper Range
NF-1	3.01	3.06	0.17	0.0559		
NF-2	3.29				2.72	3.40
NF-3	2.88					
POM-0.2-1	3.20	3.22	0.13	0.0395		
POM-0.2-2	3.07				2.96	3.47
POM-0.2-3	3.38					
POM-0.4-1	3.84	3.42	0.33	0.0971		
POM-0.4-2	3.38				2.75	4.08
POM-0.4-3	3.03					
POM-0.6-1	4.09	3.68	0.29	0.0795		
POM-0.6-2	3.48				3.09	4.26
POM-0.6-3	3.46					
POM-0.8-1	3.17	3.49	0.24	0.0680		
POM-0.8-2	3.74				3.01	3.96
POM-0.8-3	3.55					
POM-1-1	3.48	3.42	0.17	0.0507		
POM-1-2	3.59				3.07	3.76
POM-1-3	3.18					

**Table 11:** Results of flexural tests of SWSSC with different mixes.

Number	*f_f_* (MPa)	*f_f-mean_* (MPa)	Standard Deviation	COV	95% Confidence Interval	
					Lower Range	Upper Range
NF-1	6.78	6.48	0.24	0.0372		
NF-2	6.19				6.00	6.97
NF-3	6.48					
POM-0.2-1	6.49	6.56	0.09	0.0133		
POM-0.2-2	6.68				6.38	6.73
POM-0.2-3	6.50					
POM-0.4-1	6.72	6.92	0.42	0.0602		
POM-0.4-2	7.50				6.88	7.28
POM-0.4-3	6.54					
POM-0.6-1	7.22	7.08	0.10	0.0140		
POM-0.6-2	7.02				6.88	7.28
POM-0.6-3	7.00					
POM-0.8-1	6.91	6.87	0.11	0.0155		
POM-0.8-2	6.97				6.65	7.08
POM-0.8-3	6.72					
POM-1-1	6.27	6.54	0.19	0.0291		
POM-1-2	6.64				6.16	6.92
POM-1-3	6.70					

**Table 12:** Complete stress–strain curve equations for concrete under axial compression.

Equation Type	Source	Equation	
Polynomial equation	Proposed by Zhenhai Guo [62]	$y=\{\begin{array}{c} Ax+\left(3-2A\right)x^{2}+\left(A-2\right)x^{3},0\leq x\leq 1\\ \frac{x}{B{\left(x-1\right)}^{2}+x},x\geq 1 \end{array}$	(13)
Exponential equation	Proposed by Umenura [62]	$y=c\left(e^{ax}-e^{bx}\right)$	(14)
Rational fraction equation	Proposed by Sargin [62]	$y=\frac{c_{1}x+(c_{2}-1)x^{2}}{1+(c_{1}-2)x+c_{2}x^{2}}$	(15)
	Proposed by Al-Hassani [63]	$y=\{\begin{array}{c} \frac{Ax}{1+\left(2A-3\right)x^{2}+\left(2-A\right)x^{3}},0\leq x\leq 1\\ \frac{x}{B{\left(x-1\right)}^{2}+x},x\geq 1 \end{array}$	(16)
	Proposed by Saenz [64]	$y=\frac{x}{c_{1}+c_{2}x+c_{3}x^{2}+c_{4}x^{3}}$	(17)

Note: $x=\varepsilon /\varepsilon_{c}$, $y=\sigma /f_{c}$.

**Table 13:** Fitting results of parameters.

Study	Equation Coefficient	NF	POM-0.2	POM-0.4	POM-0.6	POM-0.8	POM-1
Zhenhai Guo [62]	*A*	1.078	1.238	1.672	1.814	1.153	2.385
	*R* ^2^	0.9973	0.9902	0.9978	0.9846	0.9925	0.9932
	*B*	4.113	0.7258	1.27	1.134	1.523	0.8837
	*R* ^2^	0.9906	0.9924	0.9545	0.9322	0.9963	0.9714
Umenura [62]	*a*	−0.3172	−0.7386	−0.9495	−0.9203	−0.8827	−0.5342
	*b*	−0.3015	−0.7783	−0.9842	−0.9679	−0.9181	−1.561
	*c*	−90.75	49.44	70	50.08	59.27	2.568
	*R* ^2^	0.9802	0.9644	0.9601	0.9513	0.8998	0.9816
Sargin [62]	*c*_1_	1.237	1.016	1.435	1.521	0.9915	2.165
	*c*_2_	0.2128	1.025	0.7549	0.7589	0.9057	0.7199
	*R* ^2^	0.9979	0.9804	0.9937	0.9838	0.9951	0.9893
Al-Hassani [63]	*A*	1.319	1.346	1.526	1.617	1.365	1.871
	*R* ^2^	0.9962	0.9922	0.9982	0.9939	0.9865	0.9937
	*B*	4.113	0.7258	1.27	1.134	1.523	0.8837
	*R* ^2^	0.9906	0.9924	0.9545	0.9322	0.9963	0.9714
Saenz [64]	*c*_1_	0.7607	0.6715	0.5851	0.7878	1.076	0.493
	*c*_2_	0.2946	0.2314	0.2591	−0.7334	−1.18	−0.0062
	*c*_3_	−0.8396	−0.215	−0.2149	0.9369	1.01	0.434
	*c*_4_	0.791	0.3183	0.351	0.01489	0.08796	0.07669
	*R* ^2^	0.9975	0.9924	0.9952	0.99	0.9979	0.9921

## Data Availability

Not applicable.
